# A pseudovirus-based platform to measure neutralizing antibodies in Mexico using SARS-CoV-2 as proof-of-concept

**DOI:** 10.1038/s41598-022-22921-7

**Published:** 2022-10-26

**Authors:** José Antonio Cruz-Cardenas, Michelle Gutierrez, Alejandra López-Arredondo, Julio Enrique Castañeda-Delgado, Augusto Rojas-Martinez, Yukio Nakamura, José Antonio Enciso-Moreno, Laura A. Palomares, Marion E. G. Brunck

**Affiliations:** 1 Tecnologico de Monterrey, Escuela de Ingeniería y Ciencias, Monterrey, México; 2 Instituto de Biotecnología, Universidad Nacional Autónoma de México, Ave. Universidad 2001. Col. Chamilpa, 62210 Cuernavaca, Morelos México; 3 Investigador Por México, CONACYT, Unidad de Investigación Biomédica de Zacatecas, IMSS, Zacatecas, México; 4 Tecnologico de Monterrey, Escuela de Medicina y Ciencias de la Salud, Monterrey, México; 5 Cell Engineering Division, RIKEN Bioresource Research Center, Tsukuba, Japan; 6 Unidad de Investigación Biomédica de Zacatecas-IMSS, Zacatecas, México; 7 Facultad de Química, Universidad Autónoma de Querétaro, Querétaro, México

**Keywords:** Microbiology techniques, Virology

## Abstract

The gold-standard method to evaluate a functional antiviral immune response is to titer neutralizing antibodies (NAbs) against a viral pathogen. This is historically performed using an in vitro assay of virus-mediated infection, which requires BSL-3 facilities. As these are insufficient in Latin American countries, including Mexico, scant information is obtained locally about viral pathogens NAb, using a functional assay. An alternative solution to using a BSL-3 assay with live virus is to use a BSL-2-safe assay with a non-replicative pseudovirus. Pseudoviral particles can be engineered to display a selected pathogen’s entry protein on their surface, and to deliver a reporter gene into target cells upon transduction. Here we comprehensively describe the first development of a BSL-2 safe NAbs-measuring functional assay in Mexico, based on the production of pseudotyped lentiviral particles. As proof-of-concept, the assay is based on Nanoluc luciferase-mediated luminescence measurements from target cells transduced with SARS-CoV-2 Spike-pseudotyped lentiviral particles. We applied the optimized assay in a BSL-2 facility to measure NAbs in 65 serum samples, which evidenced the assay with 100% sensitivity, 86.6% specificity and 96% accuracy. Overall, this is the first report of a BSL-2 safe pseudovirus-based functional assay developed in Mexico to measure NAbs, and a cornerstone methodology necessary to measure NAbs with a functional assay in limited resources settings.

## Introduction

Humoral immunity provides critical protection against viruses, including memory contributing to prevent future infections. In particular, neutralizing antibodies (NAbs) specifically target epitopes on viral membrane proteins, interfering with cell receptor binding^1^. Measuring the neutralizing potential of serum samples against a viral pathogen informs on effective virus-specific humoral immunity. This is relevant to: 1-monitor levels of partial protection within an asymptomatic population, 2-evaluate the humoral immunity efficacy of existing and novel vaccines against emerging pathogens, 3-test prospective therapeutic monoclonal NAbs and, overall, 4-contribute to understand immunity against viral pathogens.

Despite various viral pandemics affecting Mexico, such as the influenza caused by H1N1 and COVID-19 caused by SARS-CoV-2, no functional assay has been developed on-site to allow timely measurements of NAbs using a functional assay. For example, all currently published SARS-CoV-2 NAbs data from the Mexican population have been obtained either internationally using original virus in BLS-3 laboratories^2^, or nationally after importation of a surrogate ELISA-based kit^3–5^. Both alternatives are lengthy, expensive, and restricted by reagents availability and ongoing collaborations, respectively. This in turn could hamper the timely evaluation of the effectiveness of the national vaccination program and the monitoring of seroprevalence, both important aspects in the control of COVID-19.

In this context, to measure NAbs using a functional assay, serum samples are co-incubated with SARS-CoV-2 viral particles (VP) in a BSL-3 laboratory, after which the ability of these VP to infect target cells in vitro is measured^6^. A serum’s NAb titer negatively correlates with the level of VP-mediated infection. An alternative strategy to using authentic virus is to produce non-replicative VP that express the SARS-CoV-2 Spike (S) or RBD on their surface and that include a reporter gene delivered to target cells upon transduction^7–9^. These pseudotyped VP can then be used in BSL-2, and have been widely used to measure NAbs against a range of potentially fatal viruses, including influenza (H7N9), MERS-CoV, HCV, and SARS-CoV-2 and recent variants^10–13^. Advantages of using pseudotyped VP to measure NAbs, include their possible use in lower biosafety containment level, the facility of upscaling for high-throughput measurements at a lower cost, as well as the opportunity to customize the viral glycoprotein to match emerging variants. Here, we harness the current COVID-19 pandemic as a proof-of-concept, to develop a BLS-2-safe, functional SARS-CoV-2 NAbs titering assay in Mexico. The assay is based on non-replicative SARS-CoV-2 S pseudotyped lentiviral particles integrating Nanoluc Luciferase (Nluc) into transduced cells genomes, and all steps of development are sequentially described. The assay facilitated quantification of effective humoral immunity to SARS-CoV-2 in COVID-19 convalescent patients and BNT162b2 vaccinated individuals, and was validated against a commercially available surrogate ELISA assay, commonly used in currently published Mexican studies.

## Results

### Development and production of a SARS-CoV-2 pseudotyped lentivirus

**Figure 1:**
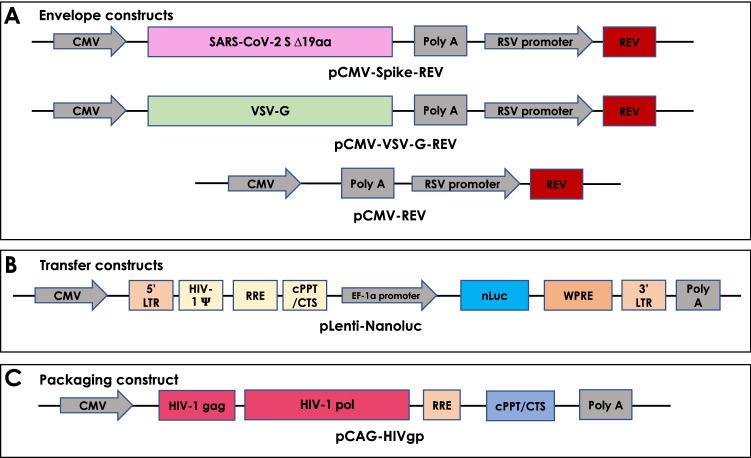
Schematic representation of constructs developed as part of a third-generation lentiviral-based system to produce VP pseudotyped using either SARS-CoV-2 S, VSV-G or no-envelope protein encoding Nluc. (**A**) Envelope, (**B**) packaging, and (**C**) transfer constructs.

To develop a BSL-2-ready assay to investigate neutralizing antibodies to SARS-CoV-2 in Mexico, we first produced SARS-CoV-2 S-pseudotyped VP. We optimized a previously reported 3rd-generation lentiviral system (Fig. 1) by using the reporter gene Nluc which is more stable and provides 100 × brighter luminescence compared to fLuc^5,9^. SARS-CoV-2 pseudotyped lentiviral vectors were produced by incorporating a SARS-CoV-2 S sequence that lacks the last 19 amino acids at the C-terminal, to increase its incorporation into pseudoviral membranes compared to the original sequence (Fig. 1A)^14^. The Nluc gene was cloned into the transfer plasmid within LTRs to allow efficient integration in target cells upon viral entry (Fig. 1B). Additional plasmids were produced for packaging VP (Fig. 1C) and as envelope controls, to express either the glycoprotein of VSV virus (VSV-G) which receptor is the ubiquitously expressed low-density lipoprotein (LDL), or no-glycoprotein (Fig. 1A)^7,12^. The integrity of all the constructions used in this work was verified by Sanger sequencing with 100% identity (Sup. Fig. 1).

**Figure 2:**
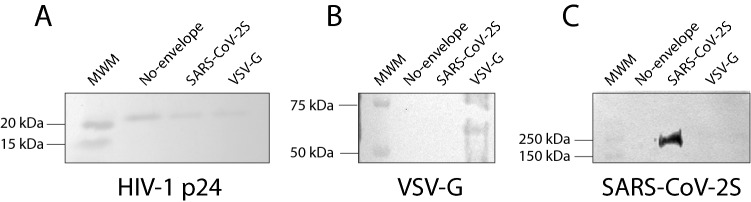
Western blot analysis of produced VP. (**A**) The structural protein p24 was detected on the 3 types of produced VP. (**B**) VSV-G was selectively detected on VSV-G VP but not on either SARS-CoV-2 S or no-envelope protein VP. (**C**) The S protein was detected as a 170 kDa protein on SARS-CoV-2 VP using a chimeric monoclonal antibody, but not on VSV-G VP. Detection of viral proteins was performed twice, of which one is shown. Original blots are presented in Sup. Fig. 2.

The selective expression of relevant viral proteins in produced VP was investigated using western blots. The presence of the structural protein p24, core component of the lentiviral particles, was confirmed in the 3 types of VP produced in this work (Fig. 2A)^13^. A specific band of size between 50 and 75 kDa was observed selectively in the VSV-G VP sample, which is consistent with the expected size of VSV-G (Fig. 2B)^15^. The selective incorporation of the S protein in SARS-CoV-2 S VP was confirmed with the detection of single band of size between 150 and 250 kDa, consistent with the expected size of 150–180 kDa, while in the same blot, no signal was detected for either the no-envelope or the VSV-G expressing VP (Fig. 2C)^9^.

### Optimization of a SARS-CoV-2 pseudovirus-based neutralization assay

**Figure 3:**
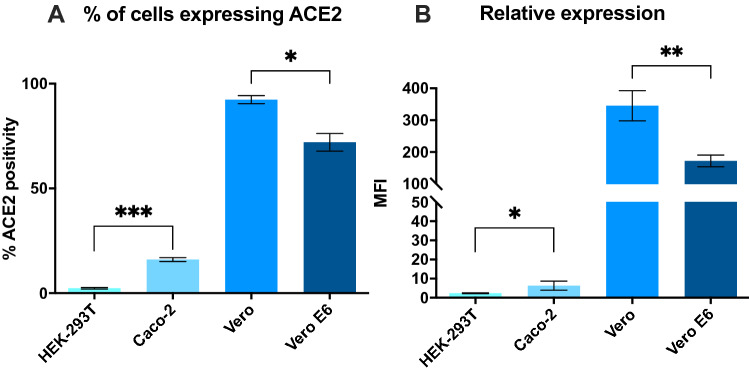
Expression of ACE2 on the surface of HEK-293 T, Caco-2, Vero, and Vero E6 cells. (**A**) Proportion of cells expressing ACE2 (**B**) Relative expression of ACE2 on cell surface as expressed by median fluorescence intensity (MFI) of ACE2-AF647. Average of 3 separate experiments done in duplicate with standard deviations shown. p < 0.05 (*), p < 0.01 (**), p < 0.001 (***).

ACE2 expression on cell surfaces correlates with SARS-CoV-2 infection susceptibility in vitro^16^. Therefore, we sought to select the most appropriate target cell line for the transduction assay by investigating ACE2 expression on exposed cell membranes of various common cell lines known to endogenously express ACE2 previously reported as target cells for SARS-CoV-2 and pseudotyped VP^12,17–19^. Caco-2 showed 16% of ACE2 positivity in culture, while Vero and Vero E6 were homogeneously highly expressing cells with 92% and 73% positivity, respectively (Fig. 3A). Heterogeneity in ACE2 expression was recently reported between single cells of various cell lines, with expression being modulated during culture and regulated epigenetically^20^. Here, Vero cells expressed a significantly higher concentration of ACE2 on cell surfaces compared to all other cell lines tested while HEK-293 T lacked expression of ACE2 (Fig. 3B), as previously described^21^. Surface expression of ACE2 suggested Vero cells to be the most susceptible to transduction by SARS-CoV-2 pseudotyped-VP. In addition to flow cytometry, various techniques have been applied with mixed results to investigate ACE2 expression by target cells, including western blot and qRT-PCR, both precluding distinction between membrane-displayed and cytosolic stores of ACE2 in contrast with flow cytometry^22,23^. However, as additional membrane proteins such as TMPRSS2 and neuropilin-1 have been evidenced as SARS-CoV-2 VP entry facilitators, the expression of ACE2 may not be sufficient on its own to predict susceptibility to infection^8,20,24^.

**Figure 4:**
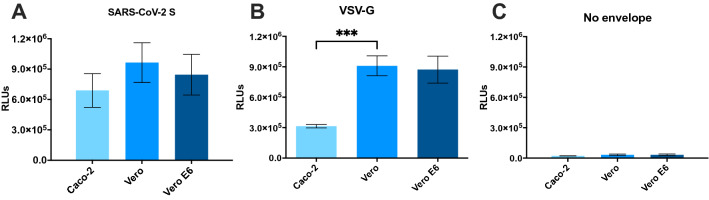
Transduction levels in Caco-2, Vero, and Vero E6 cells 24 h post-inoculation with VP, as evidenced by luminescence in relative light units (RLUs) caused by Nluc digestion of a furimazine substrate. The 3 cell lines were infected with 140 pg VP pseudotyped using (**A**) SARS-CoV-2 S, (**B**) VSV-G, or (**C**) no envelope glycoprotein. Results represent the average of 3 separate experiments performed in duplicate, and standard deviations of these results are shown, p < 0.001 (***).

Accordingly, to compare transduction susceptibility of ACE2-expressing cell lines by SARS-CoV-2 pseudotyped-VP, we co-cultured Caco-2, Vero and Vero E6 with SARS-CoV-2 pseudotyped-VP for 24 h, and measured Nluc activity as a surrogate marker for transduction. Vero cells produced the highest RLUs (mean = 9.5 × 10^5^, 8.4 × 10^5^ and 6.8 × 10^5^ RLUs for Vero, Vero E6 and Caco-2, respectively, Fig. 4A), consistent with high ACE2 expression on cell surfaces (Fig. 3). Vero and Vero E6 exhibited similar susceptibility to VSV-G with an average of 9 × 10^5^ and 8.7 × 10^5^ RLUs, respectively. On the other hand, Caco-2 showed a third of the Vero lines response (mean = 3.1 × 10^5^ RLUs, Fig. 4B). Interestingly, a previous report using a VSV-pseudotyped VP system showed Caco-2 had similar susceptibility to VSV-G VP and SARS-CoV-2 VP^8^. However, the complete SARS-CoV-2 S sequence was used in this case, while here the use of a SARS-CoV-2-S Δ19aa sequence increases the incorporation of S in VP membranes, statistically augmenting opportunities for ACE2 binding and cell entry. This is a possible cause for the reported differences in VSV-G-mediated and SARS-CoV-2-S Δ19aa-mediated transduction of Caco-2 cells. Culturing cell lines together with VP that lacked surface glycoprotein led to negligeable transductions (Fig. 4C), compared to transductions with SARS-CoV-2 S VP, with RLUs consistently < 3 × 10^4^, as expected^25^. Observed basal levels of non-specific viral entry have been reported and may be a consequence of endocytosis^21^. In addition, we produced HEK-293 T cells constitutively expressing ACE2, to demonstrate that transduction of SARS-CoV-2 S pseudotyped VP was dependent on ACE2 (Sup. Fig. 3). Overall, we replicated previous findings confirming Vero cells are highly susceptible to transduction with SARS-CoV-2 VP, leading further experiments to be performed with the Vero cell line^13^. We then evidenced 24 h transduction lead to similar RLUs compared to increased incubation time, and was therefore selected for later experiments to decrease turnaround time of the assay (Sup. Fig. 4). To identify the amount of VP required to transduce 50% of the culture (TCID50), as relevant to investigate the effect of an inhibitor in biological assays, we performed a serial dilution of the SARS-CoV-2 pseudotyped-VP and applied the Reed-Muench method (Sup. Fig. 4)^26,27^. We identified 15 pg SARS-CoV-2 pseudotyped-VP were necessary to infect 50% of 25,000 Vero cells in 96-well plate at 24 h post-inoculation.

### Neutralization of SARS-CoV-2 S lentiVP by convalescent and vaccinated sera

**Figure 5:**
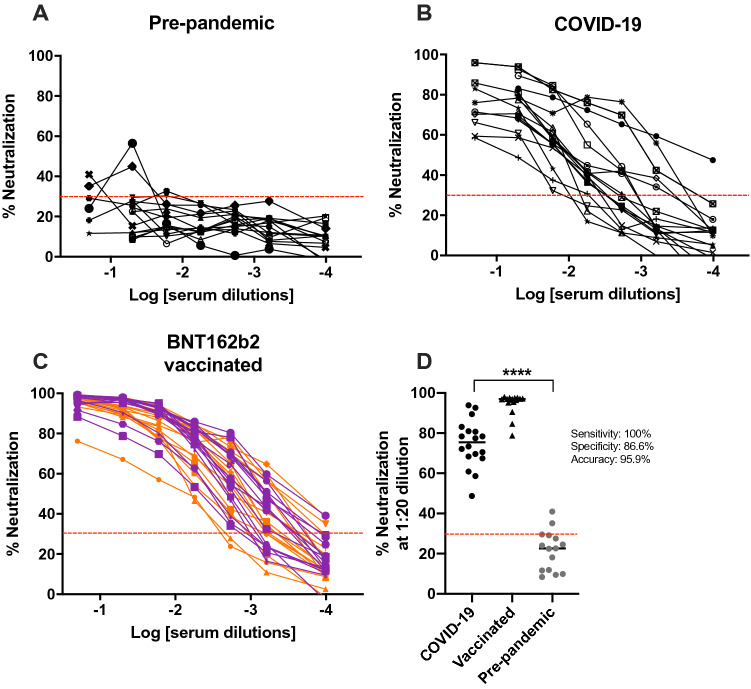
Measurement of SARS-CoV-2 S pseudotyped VP-neutralization activity from human samples. (**A**) Pre-pandemic sera. (**B**) COVID-19 convalescent sera. (**C**) BNT162b2 vaccinated sera. Orange: sample obtained on average 19 days [range: 13–22] after the first vaccine dose, purple: sample obtained on average 26 days [range: 22–29] after the 2nd vaccine dose. (**D**) Calculations of assay sensitivity, specificity and accuracy using neutralization results at 1:20 sera dilutions. The dotted line represents an arbitrary 30% neutralization cutoff for titers estimation. p < 0.0001 (****).

Once assay parameters were optimized, neutralization of transduction with human sera was implemented. Sera collected prior to the start of the COVID-19 pandemic, sera from COVID-19 diagnosed patients, and sera from healthcare professionals receiving the BNT162b2 vaccine were used as source of antibodies (Tables 1 and 2). Pre-pandemic sera showed neutralization of the SARS-CoV-2 S pseudotyped-VP ranging between 11.6% and 41% at the lowest (1:5) dilution tested and ranging between 20.2% and 4.6% at the highest tested dilution (1:9860, Fig. 5A). These results are consistent with the literature, where dilution-dependent, consistent, minimal cross-neutralization of SARS-CoV-2 VP by pre-pandemic sera are reported but considered insignificant for preventing COVID-19^7,12,28^. In another report, antibodies produced by various B cell clones obtained from a SARS-CoV 2003 outbreak survivor could efficiently neutralize SARS-CoV-2 and a SARS-related bat virus suggesting some levels of cross-neutralization^29^. All sera with prior exposure to SARS-CoV-2 (through natural infection or vaccination) could neutralize SARS-CoV-2 pseudotyped-VP efficiently. As most published pseudotyped VP-based NAb assays and the surrogate Genscript cPass ELISA assay applied a 30% neutralization cutoff to discriminate positivity, we also applied this arbitrary cutoff for consistency^2–5,30^. Convalescent COVID-19 sera were heterogeneous in their neutralization potential, with the lowest dilution (1:5) neutralizing between 95.9 and 58.5% of infection, and half of tested serum samples had a 30% neutralization titer of 540 (Fig. 5B, Table 1). Of note, the similar neutralization rates observed at the 1:5 and 1:20 dilutions (means = 76.25% and 74.7%, SD = 13. 6 and 11.6, respectively) suggest that overall NAbs contained in COVID-19 diluted down to 1:20 were in excess over VP. BNT162b2-vaccinated sera were overall systematically highly neutralizing (Fig. 5C). In contrast with convalescent sera, 14 out of 16 vaccinated samples (87.5%) had a 30% neutralization titer of 540 after the first BNT162b2 dose, and all samples had a 30% neutralization titer > 540 post-boost. Six out of the 16 individuals in the vaccinated cohort had COVID-19 positive diagnostic prior to vaccination without notable impact on reported neutralization rates (Table 2). Two individuals showed slight decrease in neutralization after the second dose (Table 2), which has been reported before^4^. Importantly, the potency of vaccinated sera was higher than COVID-19 sera, with > 18% vaccinated sera (3/16) having a 30% neutralization titer of 9860, versus only 5.5% COVID-19 sera (1/18). Using these values the presented assay has a 100% sensitivity, 86.6% specificity and 95.9% accuracy (Fig. 5D) using 1:20 serum dilution, as previously reported for such calculations^30^. Looking at individual neutralization curves, there was no consensus pattern across serum dilution (Sup. Fig. 6). Some samples exhibited similar % neutralization at the lowest dilution after the first and 2nd vaccine doses (as shown in representative samples ID 43, ID 46), while others evidenced increased neutralization at the lowest dilution after the 2nd dose (exemplified by ID 44, and to a lower extent ID 42). The shapes of neutralization curves could be concave with a slow decrease in neutralization before reaching ID50 (ID 39, ID 42, ID 21), or convex with a sharp slope around ID50 (ID 41, ID 44) and these differences could be observed within a same individual, between 2 samples (ID 43). Interestingly, the curve of convalescent patient ID 21 exhibited constant, moderately high neutralization > 75% between 1:5 and 1: 540 serum dilutions, followed by a sharp decrease in neutralization between serum dilutions 1:540 and 1:9860. Due to the shape of this curve, ID50 is extremely low suggesting very potent neutralization serum, however the patient suffered severe symptoms and passed (Table 1).

**Table 1:** Clinical parameters and neutralization information from COVID-19 convalescent sera.

ID	COVID-19 diagnostic q-PCR validated	Gender	Age	Days after initiation of symptoms at the time of sampling	Severity	Hospitalization status	Artificial respiration	Patient outcome	ID50	R2	Neutralization 1:20 sample dilution
2	Positive	M	42	0	Asx/mild	Outpatient	–	Recovered	0.001401	0.972	83.03
4	Positive	F	65	19	Asx/mild	Outpatient	–	Recovered	0.004868	0.977	69.01
7	Positive	F	76	17	Asx/mild	–	–	Recovered	0.023690	0.959	66.17
**10**	Positive	M	58	11	Asx/mild	Outpatient	–	Recovered	0.004090	0.861	70.25
**11**	Positive	M	58	30	Asx/mild	Outpatient	–	Recovered	0.006593	0.974	80.66
15	Positive	F	62	26	Severe	Severe/no UCI	–	Recovered	0.007370	0.996	67.46
18	Positive	M	53	77	Severe	Severe/no UCI	–	Recovered	0.011600	0.998	78.39
21	Positive	M	42	16	Severe	Severe/no UCI	–	Deceased	0.000000	0.976	76.02
23	Positive	M	43	18	Severe	Severe/no UCI	–	Recovered	0.018150	0.998	83.03
25	Positive	M	59	19	Severe	Severe/no UCI	–	Deceased	0.013700	0.966	58.58
**27**	Positive	M	36	9	Severe	Severe/no UCI	Yes	Deceased	0.003756	0.998	59.37
**28**	Positive	M	36	16	Severe	Severe/no UCI	Yes	Deceased	0.005466	0.935	71.41
**29**	Positive	M	36	23	Severe	Severe/no UCI	–	Deceased	0.000934	0.976	95.92
31	Positive	M	34	19	Asx/mild	Outpatient	–	Recovered	0.009537	0.944	86.85
32	Positive	F	35	21	Asx/mild	Outpatient	–	Recovered	0.001044	0.996	89.45
33	Positive	M	66	17	Severe	Severe/no UCI	–	Recovered	0.010410	0.990	85.83
34	Positive	M	52	21	Severe	Severe/no UCI	–	Recovered	0.002027	0.979	92.59
36	Positive	M	49	9	Asx/mild	Outpatient	–	Deceased	0.010780	0.983	77.48

Asx: asymptomatic. Text in bold represent longitudinal samples from same patients (patient male 58 year-old, sample IDs 10–11; Patient male 36 year-old, sample IDs 27–29).

**Table 2:** Clinical and neutralization information from BNT162b2 vaccinated individuals.

ID	COVID-19 diagnosis	Gender	First dose				Boost			
			Day post vaccine at the time of sampling post first vaccine dose	ID50	R^2^	Neutralization at 1:20 dilution	Day post vaccine at the time of sampling post second vaccine dose	ID50	R^2^	Neutralization at 1:20 dilution
39	Negative	F	16	0.001683	0.992	90.14	28	0.000554	0.986	96.9301
40	Negative	F	14	0.00226	0.999	92.78	29	0.002394	1.000	96.44067
41	Negative	F	14	0.005097	0.990	88.19	29	0.003490	0.996	84.58632
42	Positive recovered	M	14	0.001225	0.996	89.29	28	0.000724	0.999	96.60476
43	Negative	M	14	0.005883	0.988	92.08	29	0.000278	1.000	97.20769
44	Negative	F	16	0.004726	0.989	67.14	28	0.003606	1.000	90.51702
45	Negative	M	16	0.002407	0.995	93.80	28	0.001136	0.992	97.01511
46	Negative	F	16	0.001298	0.994	93.57	27	0.000439	0.999	96.9073
47	Positive recovered	M	16	0.000781	0.951	93.25	27	0.000683	0.993	95.5364521
48	Negative	F	16	0.000916	0.979	98.04	27	0.000940	1.000	97.5876662
49	Negative	F	14	0.001746	0.995	97.60	28	0.001864	0.992	97.5088973
50	Negative	F	14	0.001030	0.986	97.36	28	0.001096	0.999	98.0660083
51	Positive recovered	F	14	0.003675	0.980	92.60	28	0.001998	0.997	97.1587337
52	Positive recovered	F	13	0.001203	0.998	96.58	27	0.004783	0.997	78.7588167
53	Positive recovered	F	16	0.000000	0.998	91.51	NA	0.000967	0.975	97.5596675
54	Positive recovered	F	16	0.003309	0.981	89.43	28	0.001489	0.981	95.2267471

NA Unavailable information.

### NAb titers obtained with the developed pseudotyped VP-based assay correlate with the commercially available surrogate ELISA assay and anti-SARS-CoV-2 S total IgG concentrations

**Figure 6:**
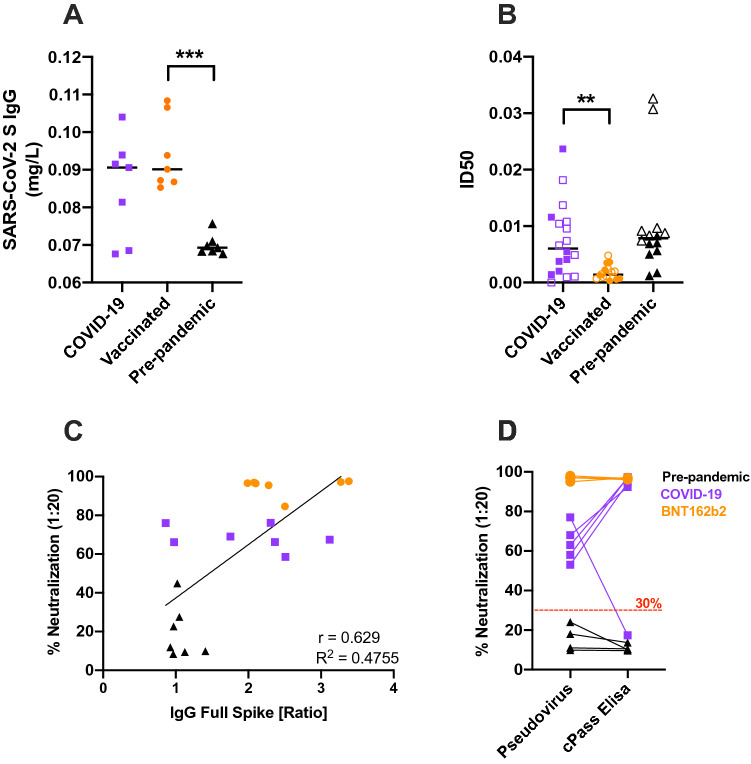
Anti-SARS-CoV-2 S total IgG underestimates neutralization potential evaluated using the SARS-CoV-2 pseudotyped VP-based assay. (**A**) SARS-CoV-2 S ELISA-based quantification of total IgG in COVID-19 convalescent sera, BNT162b2 vaccinated sera and pre-pandemic sera. (**B**) ID50 of all samples tested with SARS-CoV-2 S pseudoVP assay. Fully colored symbols indicate matched samples also tested in (**A**, **C**, **D**). (**C**) Correlation 1:20 sera neutralization potential vs. SARS-CoV-2 S IgG ratio. Purple: COVID-19 convalescent sera, orange: vaccinated sera, p < 0.001 (***) Black: pre-pandemic sera. (**D**) Comparison of COVID-19, BNT162b2 and pre-pandemic sera neutralization with pseudotyped VP-based assay and cPass SARS-CoV-2 ELISA.

To compare SARS-CoV-2 NAb and total IgG titers, 14 sera from either vaccinated (BNT162b2, 2 doses) or COVID-19 qPCR-diagnosed individuals were selected to measure total SARS-CoV-2 S IgG from samples that together exhibited a spectrum of neutralization (ranging between mild 58% to very neutralizing sera 97.5%). Total IgG against SARS-CoV-2 S1 + S2 were measured in these samples, and in 7 randomly selected pre-pandemic serum samples, using a quantitative commercial ELISA. Vaccinated and COVID-19 samples contained similar average titers of anti-SARS-CoV-2 S-specific IgG antibodies (Fig. 6A, 0.095 and 0.085 mg/L, respectively). These concentrations were on average 30% higher compared to titers measured in pre-pandemic sera (Fig. 6A). A similar increase over naïve serum concentration has been previously observed using an ELISA specific for SARS-CoV-2 S1^27^. A significant difference in total IgG concentration was evidenced between the vaccinated group and pre-pandemic group only, as the COVID-19 group presented a larger distribution of concentrations. Larger differences have been described in anti-SARS-CoV-2 S total IgG between pre-pandemic sera and sera from individuals exposed to SARS-CoV-2 antigens^28^. COVID-19 convalescent samples exhibited a wide range of ID50 while the ID50 values of BNT162b2-vaccinated samples were constricted and significantly lower (p ≤ 0.0001) (Tables 1, 2, Fig. 6B). To investigate neutralization in these samples, we used the pseudotyped VP-based neutralization assay. As evidenced earlier, an overall significantly higher neutralization was observed for both COVID-19 and vaccinated groups compared to the pre-pandemic group (p = 0.0006 for both comparisons). In addition, at dilution 1:20, there was significantly more neutralization from vaccinated samples compared to individuals exposed to the virus through infection in contrast with total IgG concentrations between these groups (Sup. Fig. 7, p = 0.0006). Higher titers of anti-SARS-CoV-2 NAb in individuals vaccinated with BNT162b2 compared to COVID-19 patients have been extensively described^4,29^. Within vaccinated samples, we could analyze a cluster of samples from volunteers who had not contracted COVID-19, evidencing a significant decrease in ID50 post-boost (Sup. Fig. 8, Table 2). In summary, after exposure to the antigen, a wide range of concentrations of anti-SARS-CoV-2 S total IgG could be measured while neutralization was restricted between 58 and 97.5% (Fig. 6C). Others have similarly evidenced a range of concentrations for total IgG between 10 and 100 mg/ml for COVID-19 patients while neutralization was constrained to > 95%^29^. To compare the results of the novel pseudotyped VP-based assay against the commonly used, commercially available, surrogate neutralization ELISA cPass assay, 5 COVID-19 convalescent sera, 5 BNT162b2 vaccinated sera and 4 pre-pandemic were tested on both platforms at the same serum dilution (Fig. 6D). All pre-pandemic samples exhibited neutralization below the positivity cutoff for both pseudotyped VP-based assay and cPass (mean neutralization %: 12.96 vs. 11.27, respectively). COVID-19 convalescent sera exhibited higher neutralization using the cPass versus the pseudotyped VP-based assay, with one COVID-19 sample showing neutralization below the 30% cutoff using cPass (17.3%), but high neutralization (77%) using the pseudotyped VP-based assay. Clinical data of the patient who provided this serum (Table 1, ID 36) indicate this patient had mild symptoms, was not hospitalized, and the sample was collected 9 days post-symptoms onset. Despite moderate ID50 (0.01078), the data indicate the patient passed. In contrast, vaccinated sera were highly neutralizing in both platforms (mean neutralization %: 96.6 and 96.8).

## Discussion

Emerging and re-emerging viral pathogens cause a huge burden on health, social welfare, and economics worldwide. However disproportioned access to resources globally has prevented relevant research in limited settings which include most Latin American countries. To date and to the best of our knowledge, no BSL2-safe functional assay has been developed in Mexico to titer NAbs for any viral pathogen. As a timely example, all currently published SARS-CoV-2 NAb data from the Mexican population have been obtained either internationally using native virus in BLS-3 facilities, or nationally after importation of the Genscript cPass™ surrogate ELISA assay. Both alternatives are lengthy, expensive, and restricted by availability and ongoing collaborations.

Scarce reports are available about SARS-CoV-2 NAb in the Mexican population, with all available data relying on a neutralization-surrogate ELISA kit^3–5^. Herein we propose a BSL-2 safe functional assay to investigate effective humoral immunity against SARS-CoV-2 locally. We produced lentiviral particles bearing SARS-CoV-2 S and optimized a highly sensitive and accurate assay of pseudotyped VP neutralization that can be deployed in most research laboratories in the country to support studies of SARS-CoV-2 induced humoral immunity.

As SARS-CoV-2 becomes endemic in human populations worldwide, various selective pressures drive the emergence of viral variants with distinct transmissibility profiles^31^. These variants in turn shape humoral immunity through specific B cell clone selection, which may compromise the efficacy of existing vaccines and increase threshold for herd immunity^32^. In addition, the half-life of SARS-CoV-2 humoral immunity, including NAbs, decays over a few months, regardless of the immunization route (natural infection or vaccination)^33,34^. Mexico has a high prevalence of comorbidities known to increase COVID-19 severity, such as obesity, diabetes and cardiovascular disease, and has experienced higher fatality rates than the global average^35–37^. Therefore, measuring NAb regularly locally is critical to monitor COVID-19 seroprevalence in Mexico.

Various pseudotyped VP-based systems have been used worldwide to assess sera- and therapeutic antibody-mediated SARS-CoV-2 neutralization, each with intrinsic protocolar and technical characteristics that have been discussed elsewhere^6,9^. Various assay parameters can be customized, affecting the results. For instance, the neutralization assay presented here has a turn-around time of 24 h after VP inoculation, while others have investigated neutralization as early as 12 h and up to 72 h^31,38^. Longer incubation times increase transduction probability, therefore comparing neutralization titers obtained using different incubation lengths could be biased^21,39,40^.

Luminescence (as a transduction surrogate marker) increases with the amount of VP applied to target cells. Accordingly, an additional factor affecting results is the differential incorporation of SARS-CoV-2 S on VP membranes, depending on the VP production technique used^12^. Using a SARS-CoV-2 sequence lacking the last 19 amino acids at the C-terminal is known to increase VP membrane incorporations^25^. Therefore, with all other parameters equal, a pseudoviral system using the authentic SARS-CoV-2 S sequence may result in lower RLUs compared to using the SARS-CoV-2 S Δ19 sequence, precluding adequate comparisons.

In this work, we used Nluc as a reporter gene with luminescence as assay read-out. This engineered and enhanced form of luciferase, provides a very sensitive assay, with reports of single-cell infections detected^9^. However, furimazine is an expensive substrate. As an alternative, others have reported the development of systems relying on fluorescence measurement to assess transduction^9,41^. These assays may not be as sensitive as luminescence-based methods but could be cheaper when applied in high-throughput screening. Adapting this assay to flow cytometry could therefore overcome the issues related to cost, and promote its usage in lower-income countries, however the sensitivity of the assays would require direct comparison and consideration.

The MOI, or amount of VP added per target cell, is also critical for reaching TCID50 within the timeframe of the assay. While some articles report volumes and dilutions of untitrated viral stock added per well, others titrate VP concentrations to provide a precise MOI^31,42^.

Target cells can be attached to wells at a pre-defined concentration at the time of adding the serum-VP mixture, or alternatively single cell suspensions of target cells may be added to the co-incubated serum-VP mixture^38^. As proteolytic cleavage of ACE2 by ADAM17 and TMPRSS2 affects susceptibility to infection, it is possible that recent trypsin treatment also impact ACE2 cleavage on target cells^43,44^. In this sense it would be relevant to compare assays with similar protocolar details for inoculation (adding VP to adherent cells, or adding freshly trypsinized cells to VP).

Finally, the neutralization threshold used to analyze results is arbitrary, with reports showing analyses using thresholds ranging between 20 and 50% neutralization^12,39,45^. This threshold is used to determine “positivity” of neutralization, and therefore affects reported neutralization titers (last dilution before neutralization curves cross the threshold), and calculated sensitivity and specificity of each assay. The aforementioned pitfalls have highlighted the need for a standardized assay to compare neutralization results across cohorts and worldwide^46^.

The literature has demonstrated the presence of cross-reactive antibodies to SARS-CoV-2 in humans. This may be due to prior infection with seasonal coronaviruses, as these share homology with SARS-CoV-2 in the S gene^47–49^. The pre-pandemic samples that were used in the present work were unfortunately not tested for antibodies against other coronaviruses so it is unclear if this is the reason for higher neutralization of these samples. Previous reports of pseudotyped virus-based neutralization assays have described their development as "sensitive" without describing a sensitivity analysis^50,51^. Others have described similar assay sensitivity to the present report^52^. A neutralization assay, based on a different system, reports sensitivity "above" 80%^53^. As the recommendation for accurate sensitivity calculations of diagnostic tests is to measure > 300 samples, we propose the present assay is sensitive, consistent with previous reports, but additional measurements are necessary to confirm precisely % sensitivity^54^.

We evidenced more variability in the magnitude of COVID-19 neutralization curves, compared to vaccinated sera. Potent NAb clones have been isolated from both high and low neutralizing titers in COVID-19 patients, which suggests SARS-CoV-2 infection hampers appropriate B cell maturation and expansion^55,56^. Much remains to be clarified about the significance of NAb titers. For instance, in COVID-19 patients, NAb titers positively correlate with disease severity^28,45,57,58^. On the other hand, a study reported that about 30% of individuals recovered from mild COVID-19 did not present NAb titers, hinting that other components of the immune system strongly contribute to recovery^55^. As evaluating T cell-mediated immunity to SARS-CoV-2 in vitro remains a challenge, monitoring NAb is mandatory to provide clues needed to elucidate immune requirements for protection against, and recovery from, SARS-CoV-2 infections. Vaccine development also requires an easily adapted and safe-to-use platform to measure the induction of immune response, in particular, the detection of NAbs generated in response to the inoculated antigen. The assay developed in this work could be adapted to emerging variants or entirely novel viral pathogens, by either applying directed mutagenesis to the S sequence, or replacing it with a synthetic gene, respectively^41^. In our hands, cloning, plasmids production and viral particles assembly and validation takes about 4 weeks. We foresee this local, cheaper, and safer platform will shed light onto the development and characterization of humoral immunity to SARS-CoV-2, and emerging viral pathogens in Mexico and Latin America.

## Material and methods

### Vector constructions

The pCAG-HIVgp and pCMV-VSV-G-RSV-REV plasmids were acquired through the Riken Institute BioResource Center^41^. The pCMV-SARS-CoV-2-S Δ19aa-RSV-REV plasmid was produced by cloning the SARS-CoV-2 S sequence, obtained from pCMV14-3X-Flag-SARS-CoV-2 S which encodes codon-optimized SARS-CoV-2 S protein lacking the last 19 amino acids at the C-terminal^25^, in place of the VSV-G gene, between the *Nhe*I and *Xba*I restriction sites. The pLenti-Nluc was produced by cloning the Nluc sequence amplified from pCCI-SP6-ZIKV-Nluc, between the *Xba*I and *Bam*HI restriction sites within the pLentiCRISPR v2 backbone (cat. 52961, Addgene, Massachussets, USA.) and removing the Cas9 gene. We produced the plasmid pCMV-REV by removing the envelope protein gene from pCMV-VSV-G-RSV-REV. pLenti-ACE2 was produced by cloning ACE2 gene obtained from pcDNA3.1-ACE2 (cat. 1786, Addgene) between the *Xba*I and XhoI restriction sites within the pLenti-EGFP plasmid, developed by our research group. All constructions were verified by restriction mapping and validated by Sanger sequencing. The datasets generated in this study are available in the GeneBank repository, accession number: ON872488 (SARS-CoV-2 S).

### Cell culture

HEK-293 T (ATCC CRL-3216), Vero (ATCC CCL-81), Vero E6 (ATCC CRL-1586) and Caco-2 (HTB-37) cells were obtained from ATCC and maintained in high-glucose DMEM (Caisson, cat. DML10) supplemented with 10% heat inactivated FBS (Sigma, Missouri, USA. cat. F2442) at 37 °C in a 5% CO_2_ atmosphere. Cells were passaged according to provider’s instructions, using either gentle scrapping or brief exposure to trypsin (Hyclone, Massachussetts, USA. cat. C838R55). All cell lines were used before passage 25.

### Production of lentiviral particles

Plasmids pLenti-Nluc, pCAG-HIVgp, and pCMV-SARS-CoV-2-S Δ19aa-RSV-REV were co-transfected at a 3:2:1 DNA ratio using the calcium phosphate method to confluent HEK-293 T cells. This transfection protocol was also used to generate the other VP used in this work (without glycoprotein or with VSV glycoprotein). For VSV-G-ACE2 pseudovirus, pLenti-ACE2, pCAG-HIVgp and pCMV-VSV-G-RSV-REV were co-transfected using the same protocol described above. Transfected cells were incubated at 37 °C with 5% CO_2_ for 24 h before replacing the medium to DMEM with 10% FBS. VP-containing supernatant was collected at 72 h post-transfection, clarified by centrifugation, filtered through a 0.45 μm PES filter (GE Healthcare, cat. 67802504), aliquoted and stored at − 80 °C until use. For each production batch, one aliquot was titrated after a single freeze–thaw cycle using the QuickTiter Lentivirus Titer Kit (Cell Biolabs, California, USA. cat. VPK-107) according to the manufacturer's protocol.

### Flow cytometry

ACE2 expression was measured on all cell lines by flow cytometry using a FACS Celesta fitted with 405 nm, 488 nm and 640 nm lasers (BD Biosciences). Briefly 3 × 10^5^ cells were incubated on ice with mouse anti-human ACE2 monoclonal antibody conjugated to AF-647 (Santa Cruz Biotechnology, USA, cat. SC-390851) following manufacturer’s instructions. Propidium iodide (BD Biosciences, cat. 51-6621E) was added 15 min before acquisition on the flow cytometer, as per manufacturer’s instructions. At least 20,000 events were acquired per sample, and the data was analysed using FlowJo v.10.

### Transduction assay

Transductions were performed over 24 h (Sup. Fig. 4). Transduction of target cells by VP was assessed by luminescence or flow cytometry, according to the nature of the transgene in the transfer plasmid. For Nanoluc luciferase, transduction efficacy was assessed by measuring luminescence intensity, induced by the conversion of furimazine, reported in RLUs. RLUs were measured using a commercial kit according to manufacturer’s instructions (Promega, USA. cat. N1110), including reading at 460 nm on a Biotek Synergy Microplate Reader. Uninfected cells were used for normalization. For VSV-G-ACE-2 transduction efficacy in HEK-293 T cells, ACE2 expression was evaluated by flow cytometry. Briefly, 3 × 10^5^ cells were stained with a mouse anti-human ACE2 monoclonal antibody conjugated to AF-647 (Santa Cruz Biotechnology, USA, cat. SC-390851) following manufacturer’s instructions. Twenty thousand events were acquired and analyzed in FlowJo v.10.

### Generation of HEK-293 T stably expressing ACE2

To generate the HEK-ACE2 cell line, 5 × 10^5^ HEK-293 T cells were infected with 150 pg of VSV-ACE2 pseudovirus in a 6-well plate with a total volume of 1.5 ml. Validation of transduced pool was performed by measuring the ACE2 expression by flow cytometry following the protocol described above. ACE2 positive cells were sorted out using a FACS Melody (BD Biosciences). Single clones were expanded and stored until their use.

### Western blot

The selective incorporation of SARS-CoV-2-S or VSV-G, and p24 proteins in VP was validated by western blot. Briefly, supernatants were pelleted by ultra-centrifugation at 100,000*g* for 5 h over a 20% sucrose cushion. The supernatant was removed, and the viral pellet was resuspended in 60 μl of PBS. Thirty μl of each sample was subjected to SDS-PAGE followed by immunoblotting on PVDF membranes. Mouse anti-VSV-G-HRP (Santa Cruz, cat. SC-365019-HRP) and mouse anti-HIV1-p24-HRP (Santa Cruz, cat. 69728-HRP) were used for one-step detection. The chimeric monoclonal antibody 20n01 specific against SARS-CoV-2 S was kindly donated by the laboratory of Dr. Jesús Hernandez (CIAD, Mexico)^59^ and used as a primary antibody, followed by a rabbit anti-human IgG HRP (Abcam, cat. ab6759). Membranes were revealed using the Immobilon Forte Western HRP substrate (Millipore, cat. WBLUF0500) following the manufacturer’s protocol. Chemiluminescence signal was acquired through iBright 1500 (Invitrogen).

### TCID50 determination

TCID50 assays were performed as previously described^3^. Viral stocks were serially diluted threefold and each dilution assessed in 6 replicates in the transduction of 25,000 Vero cells per well, seeded in a 96-well plate and incubated at 37 °C in a 5% CO_2_ atmosphere. Twenty-four h post-seeding, luminescence was measured as a surrogate for transduction as described above.

### Pseudovirus-based SARS-CoV-2 Spike neutralization assay

Protocols for the use of human samples for this work were approved by the IRB of the Instituto Mexicano del Seguro Social (IMSS) with reference number R-2020-785-068 prior to starting this work, and applied in accordance with the Declaration of Helsinki. A total of 15 SARS-CoV-2 free, collected between 2014 and 2018 (prior to the 2019 initial outbreak, collected through IRB approved protocols at IMSS, for bio banking purposes), and 18 COVID-19 blood samples were obtained from 15 patients (confirmed by a positive qRT-PCR diagnostic, Table 1). Samples were collected and used upon signed informed consent and anonymization. Vaccinated sera were obtained from health care professionals receiving the BNT162b2 vaccine. Blood samples were collected between 14 and 16 days post-application of the first dose, and a second sampling was performed 27 to 29 days after the application of the second dose. Status of prior infection with SARS-CoV-2 was also recorded (Table 2). Briefly, sera were enriched from coagulated blood by centrifugation, inactivated for 30 min at 56 °C, and aliquoted and stored at − 80 °C until use. On the day of the assay, sera were serially diluted 7-folds, spanning 1:5 to 1:9860, and 100 μL of each dilution was incubated for 1 h at 37 °C and 5% CO_2_, together with 15 pg of SARS-CoV-2 VP in duplicate in a 96-well plate. Post-incubation, 25,000 Vero cells were added to each well and the plate was incubated for 24 h at 37 °C and 5% CO_2_. As a positive control for transduction, SARS-CoV-2 VP were incubated with Vero cells. Vero cells seeded in triplicate were used for basal luminescence background assessment as described earlier. After 24 h, Nluc levels were measured as described above. Neutralization is described as % inhibition of transduction, calculated as:

$$\% \;neutralization = 100 - \frac{{\left( {{\text{mean}}\left[ {{\text{sample}}} \right]{\text{RLUs}}{-}{\text{mean}}\left[ {{\text{background}}} \right]{\text{RLUs}}} \right) \times 100}}{{{\text{mean}}\left[ {{\text{virus}}\;{\text{only}}} \right]{\text{RLUs}}{-}{\text{mean}}\left[ {{\text{background}}} \right]{\text{RLUs}}}}$$

All experiments using human serum samples were performed according to national and institutional regulations.

### ELISA-based neutralizing antibodies measurements

COVID-19 (samples 10, 11, 27, 28, and 36), the second dose of BNT162b2 vaccinated (samples 39, 43, 45, 51, and 54), and 2 pre-pandemic samples were used to measure and compare neutralizing activity using the cPass SARS-CoV-2 Neutralization Antibody Detection Kit (Genscript, cat. L00847). The neutralization test was performed following the manufacturer's protocol. The inhibition rate and 30% cut-off value were set according to the manufacturer's instructions.

### Statistical analysis

The distribution was investigated using the Anderson Darling test. In the case of categorical values for calculation of Sn and Sp, a contingency 2 × 2 table was used with a fisher exact test. For flow cytometry analyzes, medians of fluorescence intensity and percentage of positive cells were compared using Mann–Whitney and P values are reported. To calculate ID50, neutralization curves were log transformed, normalized, and fitted to the most appropriate model between log(inhibitor) vs. response (three parameters) and log(inhibitor) vs. response, variable slope (four parameters). For calculating sensitivity and specificity of the assay, we determined sera positivity and negativity at a final 1:20 dilution and using a 30% neutralization threshold, as previously reported to evidence true/false positives and true/false negatives^30^. All statistical analyzes were performed using GraphPad Prism v.9 software and p-values < 0.05 were considered statistically significant.

## Supplementary Information

Supplementary Figure 1.

Supplementary Figure 2.

Supplementary Figure 3.

Supplementary Figure 4.

Supplementary Figure 5.

Supplementary Figure 6.

Supplementary Figure 7.

Supplementary Figure 8.
