# Supplementary material for: A pseudovirus-based platform to measure neutralizing antibodies in Mexico using SARS-CoV-2 as proof-of-concept
Source: Sci Rep. 2022 Oct 26;12:17966. doi: 10.1038/s41598-022-22921-7 (PMC9606276; doi:10.1038/s41598-022-22921-7)
Supplement: Supplementary file 1 — Supplementary Figure 1. [file 41598_2022_22921_MOESM1_ESM.docx]

**Sup. Fig. 1:** Sequences of relevant plasmid portions obtained by Sanger sequencing.

### Spike Δ19

ATGTTCGTTTTCCTTGTTCTGTTGCCTCTCGTTAGTAGCCAATGCGTCAACCTTACTACTAGAACCCAGCTCCCTCCAGCATATACCAACTCTTTCACCAGGGGCGTATATTACCCGGACAAAGTGTTCCGCTCAAGTGTGCTGCATTCTACGCAGGACCTTTTCTTGCCCTTTTTCAGTAATGTTACTTGGTTTCATGCTATCCATGTGTCTGGAACTAACGGAACCAAGCGCTTTGACAACCCCGTCCTCCCTTTCAACGATGGCGTGTACTTCGCTTCCACGGAAAAGTCAAACATAATTCGCGGCTGGATCTTTGGTACAACACTCGACTCAAAGACGCAGAGCCTGCTGATCGTTAATAACGCTACAAATGTTGTGATAAAGGTGTGTGAATTTCAGTTCTGCAATGATCCCTTCCTGGGTGTGTACTACCATAAGAATAACAAGAGCTGGATGGAATCCGAATTTAGGGTTTACAGTTCCGCTAACAACTGCACATTCGAATACGTAAGCCAGCCATTTCTTATGGATCTTGAGGGCAAGCAAGGAAACTTCAAGAACTTGAGGGAGTTCGTGTTCAAAAATATCGACGGCTATTTTAAGATATATAGCAAGCACACTCCAATAAACTTGGTGCGCGACCTGCCCCAGGGATTCTCTGCTCTGGAGCCCCTGGTGGATCTGCCCATTGGAATAAACATAACTCGCTTTCAAACACTGCTCGCCCTGCATCGCAGTTACCTCACCCCTGGTGATAGTAGTTCAGGATGGACAGCAGGAGCCGCCGCATACTACGTCGGCTACCTGCAGCCTAGGACCTTCTTGCTGAAGTACAACGAGAACGGTACAATAACTGACGCTGTGGACTGCGCTCTGGACCCTCTGTCCGAGACGAAGTGCACCCTGAAGAGCTTTACTGTTGAAAAAGGCATTTACCAAACCAGCAACTTCCGCGTCCAGCCAACCGAGAGCATCGTCAGATTTCCCAACATTACAAATCTGTGTCCCTTCGGCGAGGTGTTCAACGCCACACGCTTCGCTTCAGTGTACGCATGGAACCGCAAGCGCATATCTAACTGCGTCGCGGATTATTCTGTCCTCTACAACTCCGCCTCTTTCTCCACCTTCAAGTGCTACGGAGTGTCACCGACTAAGCTGAACGATCTCTGCTTTACCAACGTCTACGCGGACTCCTTCGTGATAAGAGGTGATGAAGTGAGACAAATAGCCCCAGGTCAGACTGGTAAGATCGCAGATTACAACTACAAATTGCCTGATGATTTCACTGGTTGCGTTATCGCGTGGAACTCTAATAACCTCGATTCTAAGGTCGGTGGTAACTACAATTACCTGTACCGCTTGTTTAGGAAGTCAAACCTGAAGCCTTTCGAGAGGGATATTTCAACCGAAATCTATCAAGCGGGTTCAACACCGTGTAACGGTGTGGAAGGATTTAACTGCTACTTCCCCCTGCAGTCTTACGGATTCCAGCCAACCAATGGCGTGGGTTACCAACCTTATCGCGTGGTGGTTCTGAGTTTCGAACTGTTGCACGCTCCCGCCACGGTATGCGGTCCCAAGAAGAGCACTAACTTGGTGAAGAATAAGTGCGTGAATTTCAATTTCAATGGCCTCACTGGAACTGGAGTGCTGACCGAATCCAATAAGAAGTTCTTGCCCTTCCAGCAGTTCGGAAGAGACATTGCTGACACAACCGACGCGGTGCGCGATCCTCAGACTCTGGAGATATTGGACATTACACCATGTTCTTTCGGCGGTGTGTCTGTCATTACTCCGGGCACGAATACTAGCAACCAGGTAGCCGTGCTGTACCAAGACGTGAATTGCACAGAGGTTCCCGTCGCAATTCACGCTGACCAGCTGACCCCCACGTGGAGGGTTTACAGCACTGGTAGTAACGTCTTCCAGACGAGAGCCGGTTGCTTGATCGGAGCGGAACATGTGAATAACTCCTACGAGTGCGACATCCCCATCGGAGCCGGTATATGCGCCTCTTATCAGACACAAACTAACTCACCCAGGAGAGCCCGCAGTGTGGCTTCTCAAAGCATTATAGCATACACTATGTCTCTTGGTGCCGAAAATTCCGTGGCCTATTCTAACAATTCAATCGCCATCCCAACCAACTTCACAATTAGCGTGACTACCGAAATACTGCCTGTGAGCATGACGAAAACCAGCGTAGACTGCACTATGTATATCTGTGGAGACTCCACTGAGTGCTCCAACCTTCTCCTGCAGTACGGTAGCTTCTGTACCCAATTGAACCGCGCCCTTACAGGCATCGCTGTTGAGCAAGATAAGAATACCCAGGAAGTTTTTGCCCAGGTTAAGCAGATATACAAAACACCGCCCATTAAGGACTTCGGAGGCTTCAACTTCTCTCAGATACTGCCTGACCCCTCCAAGCCATCAAAACGCAGCTTCATTGAGGACCTCTTGTTCAACAAAGTGACTCTGGCTGATGCTGGCTTCATTAAGCAGTACGGAGATTGCCTGGGAGATATTGCTGCCAGGGACCTCATCTGCGCCCAGAAGTTTAATGGCCTGACAGTCTTGCCCCCACTTCTGACAGACGAGATGATTGCTCAGTACACATCTGCCCTCCTCGCTGGCACCATAACATCCGGATGGACATTTGGTGCTGGTGCTGCCCTCCAGATTCCCTTCGCAATGCAGATGGCGTATCGCTTTAACGGCATCGGTGTCACACAAAACGTGTTGTATGAGAACCAAAAGCTCATCGCTAACCAGTTTAATTCTGCTATTGGTAAGATTCAGGACAGCCTGTCATCAACCGCGTCTGCCCTTGGTAAGTTGCAGGACGTGGTGAACCAGAATGCTCAGGCTTTGAATACTCTGGTGAAGCAACTCTCTTCAAATTTCGGCGCTATCTCTTCTGTGTTGAACGACATCCTGAGTCGCCTTGATAAGGTGGAAGCTGAAGTTCAAATTGATAGATTGATTACTGGCAGGCTCCAGTCTTTGCAGACCTACGTTACACAGCAGCTGATTAGGGCGGCTGAAATTAGAGCTTCCGCCAATCTGGCTGCAACCAAGATGTCCGAATGCGTCCTGGGTCAGTCAAAGCGCGTTGACTTTTGTGGTAAAGGCTACCACCTCATGTCATTTCCCCAGTCAGCACCTCACGGAGTAGTGTTCCTCCACGTCACCTACGTTCCAGCACAGGAAAAGAATTTTACCACTGCGCCGGCAATCTGTCACGACGGTAAGGCACACTTCCCCCGCGAGGGCGTATTCGTGTCTAACGGAACTCATTGGTTCGTCACACAGAGAAACTTCTATGAGCCTCAGATCATTACCACCGACAATACATTTGTGTCCGGTAACTGCGACGTTGTGATTGGAATCGTCAACAACACTGTGTACGATCCACTTCAGCCAGAACTGGATAGCTTCAAGGAAGAATTGGACAAATATTTCAAAAATCACACTTCACCCGATGTGGACCTGGGTGACATTAGTGGTATCAATGCGTCCGTGGTCAATATTCAAAAAGAGATTGACAGGCTCAACGAAGTGGCCAAGAACCTGAACGAAAGTCTTATCGATCTGCAAGAATTGGGAAAGTATGAGCAGTACATCAAGTGGCCGTGGTACATTTGGTTGGGTTTTATCGCCGGTCTGATCGCCATCGTTATGGTTACCATTATGCTTTGCTGCATGACGAGCTGTTGCTCCTGTCTGAAGGGATGCTGCTCTTGCGGATCATGTTGC

### Rev gene

ATGGCAGGAAGAAGCGGAGACAGCGACGAAGACCTCCTCAAGGCAGTCAGACTCATCAAGTTTCTCTATCAAAGCAACCCACCTCCCAATCCCGAGGGGACCCGACAGGCCCGAAGGAATAGAAGAAGAAGGTGGAGAGAGAGACAGAGACAGATCCATTCGATTAGTGAACGGATCCTTAGCACTTATCTGGGACGATCTGCGGAGCCTGTGCCTCTTCAGCTACCACCGCTTGAGAGACTTACTCTTGATTGTAACGAGGATTGTGGAACTTCTGGGACGCAGGGGGTGGGAAGCCCTCAAATATTGGTGGAATCTCCTACAATATTGGAGTCAGGAGCTAAAGAATAG

### 5’ LTR

GGGTCTCTCTGGTTAGACCAGATCTGAGCCTGGGAGCTCTCTGGCTAACTAGGGAACCCACTGCTTAAGCCTCAATAAAGCTTGCCTTGAGTGCTTCAAGTAGTGTGTGCCCGTCTGTTGTGTGACTCTGGTAACTAGAGATCCCTCAGACCCTTTTAGTCAGTGTGGAAAATCTCTAGCA

### 3’ LTR

TGGAAGGGCTAATTCACTCCCAACGAAGACAAGATCTGCTTTTTGCTTGTACTGGGTCTCTCTGGTTAGACCAGATCTGAGCCTGGGAGCTCTCTGGCTAACTAGGGAACCCACTGCTTAAGCCTCAATAAAGCTTGCCTTGAGTGCTTCAAGTAGTGTGTGCCCGTCTGTTGTGTGACTCTGGTAACTAGAGATCCCTCAGACCCTTTTAGTCAGTGTGGAAAATCTCTAGCA

### Nluc gene

ATGGTCTTCACACTCGAAGATTTCGTTGGGGACTGGCGACAGACAGCCGGCTACAACCTGGACCAAGTCCTTGAACAGGGAGGTGTGTCCAGTTTGTTTCAGAATCTCGGGGTGTCCGTAACTCCGATCCAAAGGATTGTCCTGAGCGGTGAAAATGGGCTGAAGATCGACATCCATGTCATCATCCCGTATGAAGGTCTGAGCGGCGACCAAATGGGCCAGATCGAAAAAATTTTTAAGGTGGTGTACCCTGTGGACGATCATCACTTTAAGGTGATCCTGCACTATGGCACACTGGTAATCGACGGGGTTACGCCGAACATGATCGACTATTTCGGACGGCCGTATGAAGGCATCGCCGTGTTCGACGGCAAAAAGATCACTGTAACAGGGACCCTGTGGAACGGCAACAAAATTATCGACGAGCGCCTGATCAACCCCGACGGCTCCCTGCTGTTCCGAGTAACCATCAACGGAGTGACCGGCTGGCGGCTGTGCGAACGCATTCTGGCGTAA

### RRE

AGGAGCTTTGTTCCTTGGGTTCTTGGGAGCAGCAGGAAGCACTATGGGCGCAGCGTCAATGACGCTGACGGTACAGGCCAGACAATTATTGTCTGGTATAGTGCAGCAGCAGAACAATTTGCTGAGGGCTATTGAGGCGCAACAGCATCTGTTGCAACTCACAGTCTGGGGCATCAAGCAGCTCCAGGCAAGAATCCTGGCTGTGGAAAGATACCTAAAGGATCAACAGCTCCT

### cPPT

TTTTAAAAGAAAAGGGGGGATTGGGGGGTACAGTGCAGGGGAAAGAATAGTAGACATAATAGCAACAGACATACAAACTAAAGAATTACAAAAACAAATTACAAAAATTCAAAATTTT

### WPRE

AATCAACCTCTGGATTACAAAATTTGTGAAAGATTGACTGGTATTCTTAACTATGTTGCTCCTTTTACGCTATGTGGATACGCTGCTTTAATGCCTTTGTATCATGCTATTGCTTCCCGTATGGCTTTCATTTTCTCCTCCTTGTATAAATCCTGGTTGCTGTCTCTTTATGAGGAGTTGTGGCCCGTTGTCAGGCAACGTGGCGTGGTGTGCACTGTGTTTGCTGACGCAACCCCCACTGGTTGGGGCATTGCCACCACCTGTCAGCTCCTTTCCGGGACTTTCGCTTTCCCCCTCCCTATTGCCACGGCGGAACTCATCGCCGCCTGCCTTGCCCGCTGCTGGACAGGGGCTCGGCTGTTGGGCACTGACAATTCCGTGGTGTTGTCGGGGAAATCATCGTCCTTTCCTTGGCTGCTCGCCTGTGTTGCCACCTGGATTCTGCGCGGGACGTCCTTCTGCTACGTCCCTTCGGCCCTCAATCCAGCGGACCTTCCTTCCCGCGGCCTGCTGCCGGCTCTGCGGCCTCTTCCGCGTCTTCGCCTTCGCCCTCAGACGAGTCGGATCTCCCTTTGGGCCGCCTCCCCGC
