# Supplementary material for: A pseudovirus-based platform to measure neutralizing antibodies in Mexico using SARS-CoV-2 as proof-of-concept
Source: Sci Rep. 2022 Oct 26;12:17966. doi: 10.1038/s41598-022-22921-7 (PMC9606276; doi:10.1038/s41598-022-22921-7)
Supplement: Supplementary file 2 — Supplementary Figure 2. [file 41598_2022_22921_MOESM2_ESM.pdf]

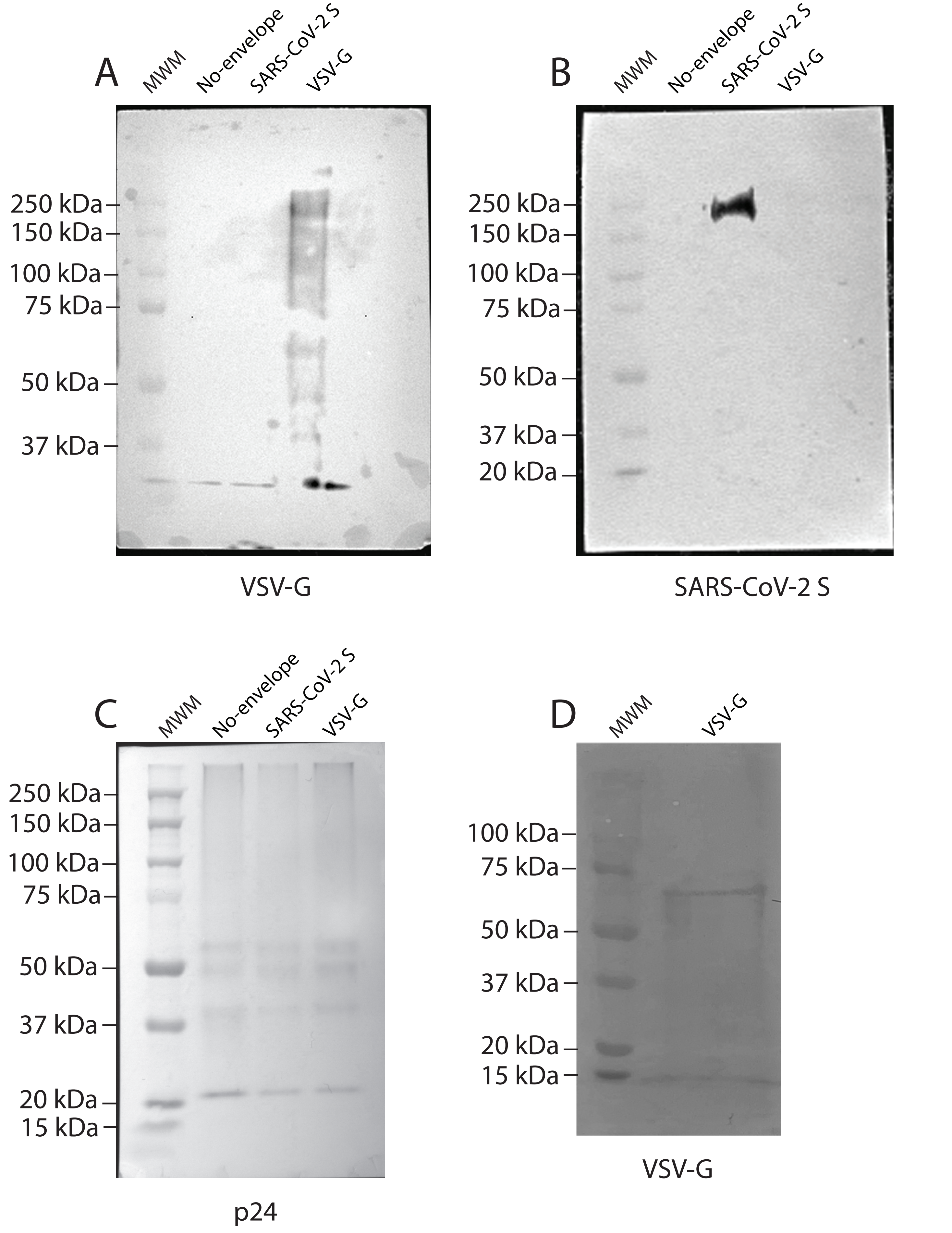

Sup. Fig. 2. Full-length blots originating Fig. 2. Original images retrieved from iBright 1500 (Invitrogen). Labels were added to indicate molecular weight marker (MWM) and samples. Each image corresponds to a different and independent experiment from where cropped images shown in Figure 2. A: VSV-G detection in VSV-G pseudotyped VP sample, B: Spike detection in SARS-CoV-2 S pseudotyped VP sample, C: p24 detection in all samples, D) VSV-G detection in VSV-G pseudotyped VP sample, colorimetric assay.
