# Supplementary material for: A pseudovirus-based platform to measure neutralizing antibodies in Mexico using SARS-CoV-2 as proof-of-concept
Source: Sci Rep. 2022 Oct 26;12:17966. doi: 10.1038/s41598-022-22921-7 (PMC9606276; doi:10.1038/s41598-022-22921-7)
Supplement: Supplementary file 3 — Supplementary Figure 3. [file 41598_2022_22921_MOESM3_ESM.pdf]

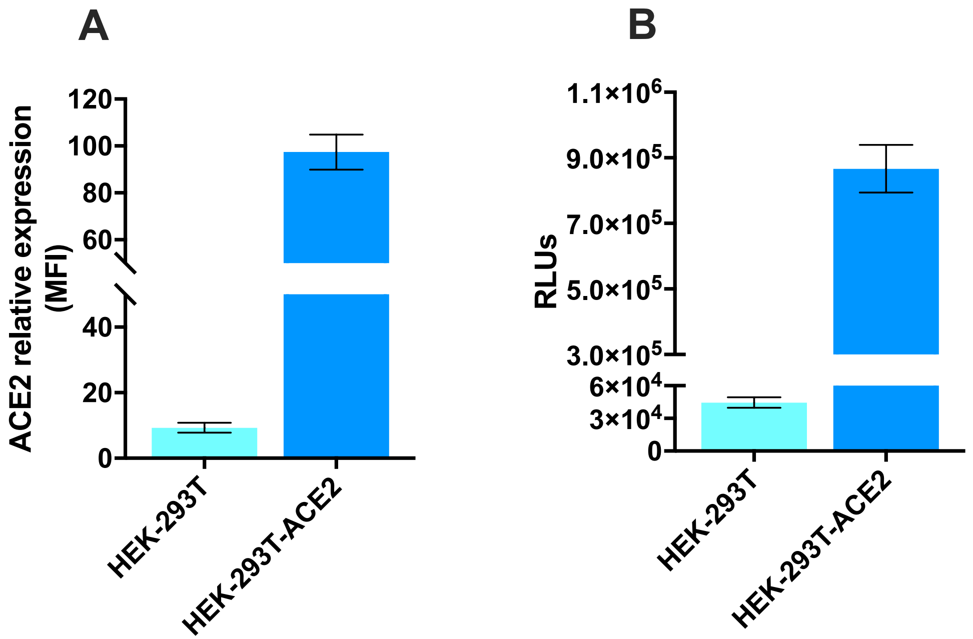

Sup. Fig. 3. SARS-CoV-2 S pseudotyped VP entry is dependent on ACE2. **A:** Relative expression of ACE2 on the surface of HEK-293T and HEK-293T-ACE2 expressed as median fluorescence intensity (MFI) as measured by flow cytometry. **B:** RLUs of HEK-293T and HEK-293T-ACE2 transduced with 140 pg of SARS-CoV-2 S pseudotyped VP. The average of an independent experiment ran in triplicate, is shown. Error bars indicate SD.
