# Supplementary material for: A pseudovirus-based platform to measure neutralizing antibodies in Mexico using SARS-CoV-2 as proof-of-concept
Source: Sci Rep. 2022 Oct 26;12:17966. doi: 10.1038/s41598-022-22921-7 (PMC9606276; doi:10.1038/s41598-022-22921-7)
Supplement: Supplementary file 4 — Supplementary Figure 4. [file 41598_2022_22921_MOESM4_ESM.pdf]

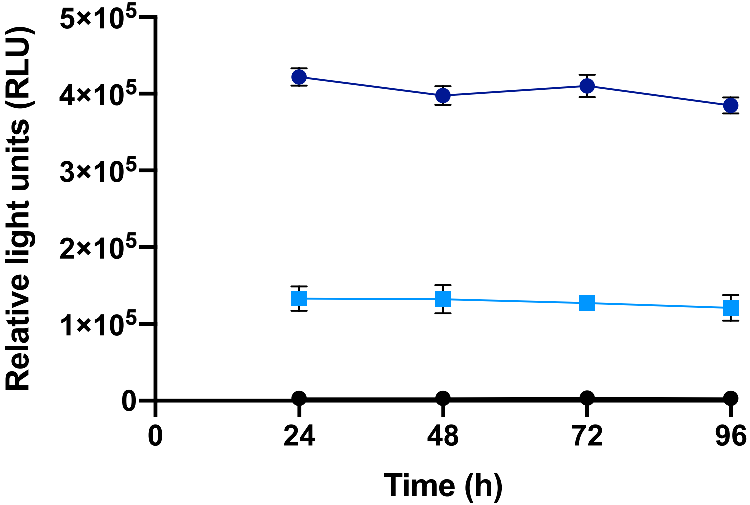

Sup. Fig. 4. Kinetics of infection of Vero cells with SARS-CoV-2-S pseudoVP. Dark blue: 12,500 cells infected with 0.035 ng of SARS-CoV-2 S pseudoVP. Light blue: 12,500 cells infected with 0.01 ng of SAR-CoV-2 S pseudoVP. Black: Background of 12,500 cells without infection.
