# Supplementary material for: A pseudovirus-based platform to measure neutralizing antibodies in Mexico using SARS-CoV-2 as proof-of-concept
Source: Sci Rep. 2022 Oct 26;12:17966. doi: 10.1038/s41598-022-22921-7 (PMC9606276; doi:10.1038/s41598-022-22921-7)
Supplement: Supplementary file 5 — Supplementary Figure 5. [file 41598_2022_22921_MOESM5_ESM.pdf]

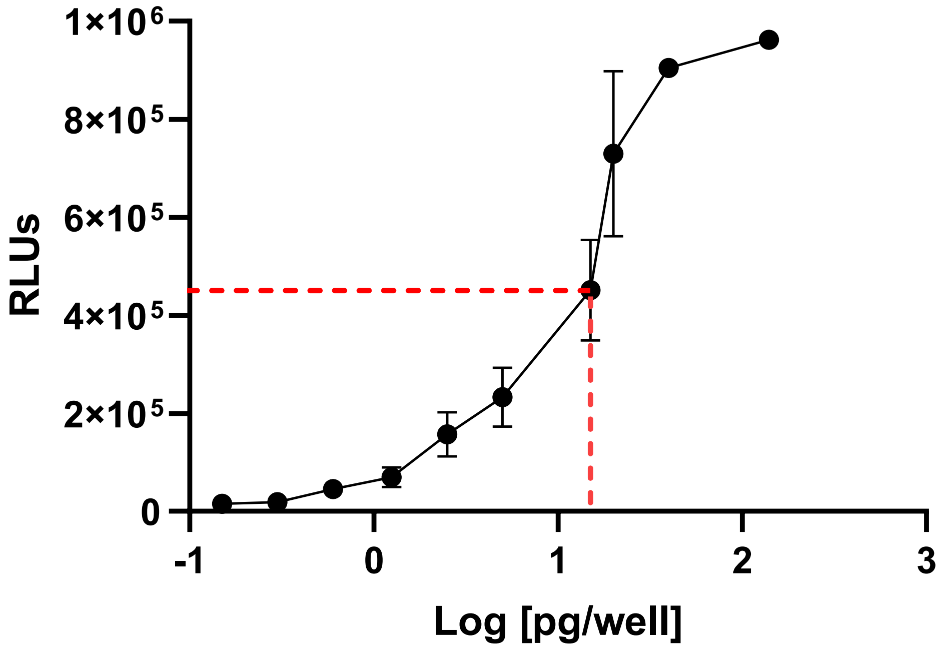

Sup. Fig. 5. Determination of the $TCID_{50}$ of SARS-CoV-2 S pseudovirus using 25,000 Vero cells as target in 96 well plate with infection assessed at 24 h. The dotted line represents 50% of maximal RLUs equivalent to 15 pg VP per well. The average of 2 independent experiments, ran in triplicate, is shown. Error bars indicate SD.
