# Supplementary material for: A pseudovirus-based platform to measure neutralizing antibodies in Mexico using SARS-CoV-2 as proof-of-concept
Source: Sci Rep. 2022 Oct 26;12:17966. doi: 10.1038/s41598-022-22921-7 (PMC9606276; doi:10.1038/s41598-022-22921-7)
Supplement: Supplementary file 6 — Supplementary Figure 6. [file 41598_2022_22921_MOESM6_ESM.pdf]

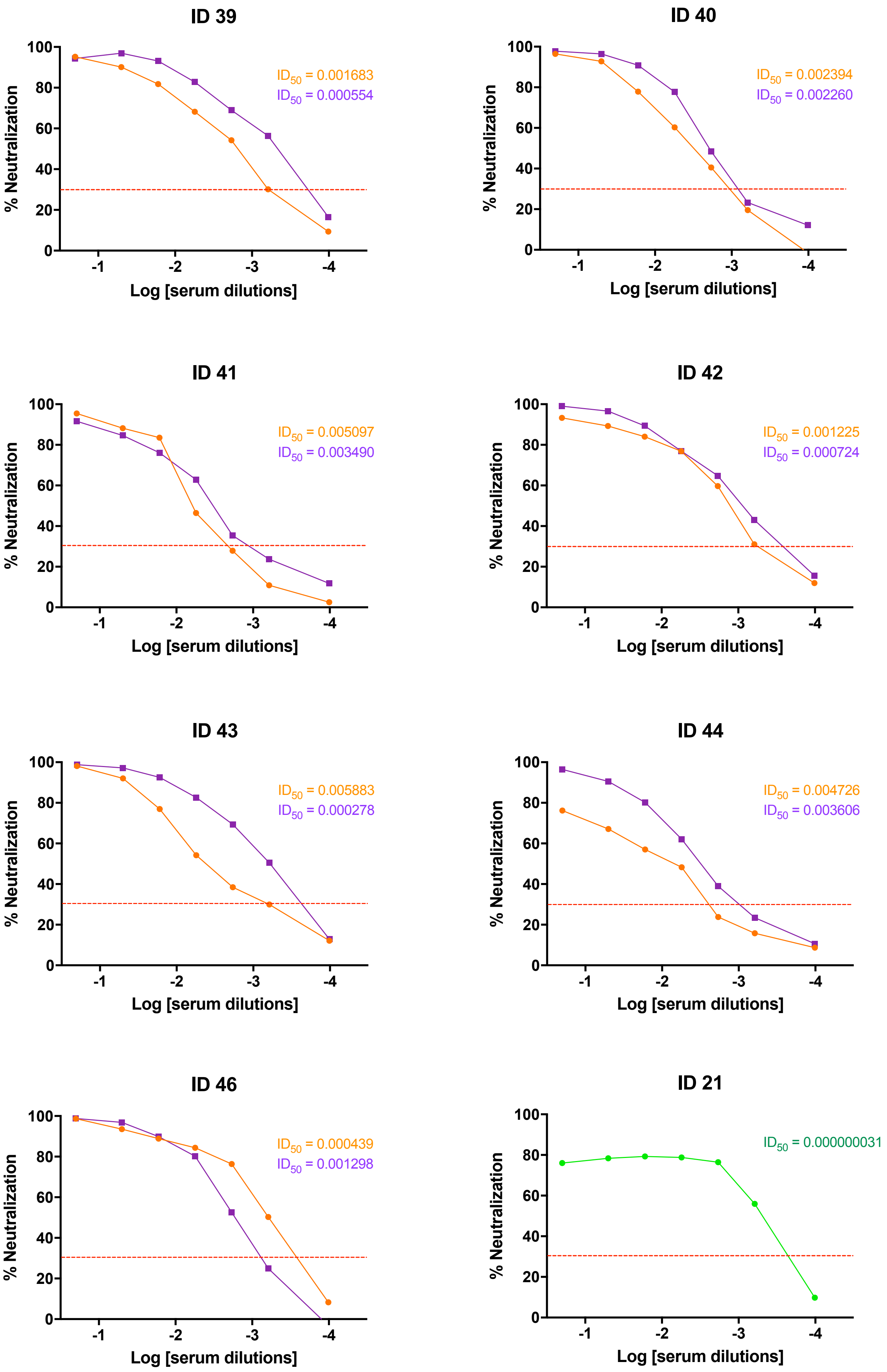

Sup. Fig. 6. Example of individual patterns of neutralization. Orange: first dose of BNT162b2 vaccine, purple: second dose BNT162b2 vaccine, green: COVID-19 sera sample. Dotted line represents an arbitrary 30% neutralization cutoff.
