# Supplementary material for: A pseudovirus-based platform to measure neutralizing antibodies in Mexico using SARS-CoV-2 as proof-of-concept
Source: Sci Rep. 2022 Oct 26;12:17966. doi: 10.1038/s41598-022-22921-7 (PMC9606276; doi:10.1038/s41598-022-22921-7)
Supplement: Supplementary file 7 — Supplementary Figure 7. [file 41598_2022_22921_MOESM7_ESM.pdf]

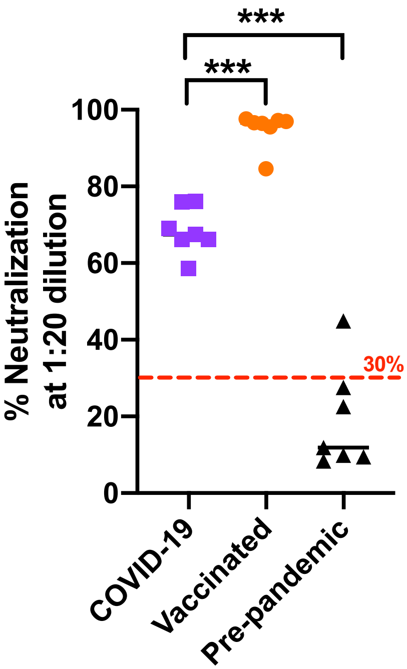


**Sup. Fig. 7.** SARS-CoV-2 S VP neutralization by matched sera (used in Fig 6A) at 1:20 dilution. Purple: COVID-19 convalescent sera, orange: second dose BNT162b2 vaccine, black: Pre-pandemic samples. Dotted line represents an arbitrary 30% neutralization cutoff.
