# Supplementary material for: A pseudovirus-based platform to measure neutralizing antibodies in Mexico using SARS-CoV-2 as proof-of-concept
Source: Sci Rep. 2022 Oct 26;12:17966. doi: 10.1038/s41598-022-22921-7 (PMC9606276; doi:10.1038/s41598-022-22921-7)
Supplement: Supplementary file 8 — Supplementary Figure 8. [file 41598_2022_22921_MOESM8_ESM.pdf]

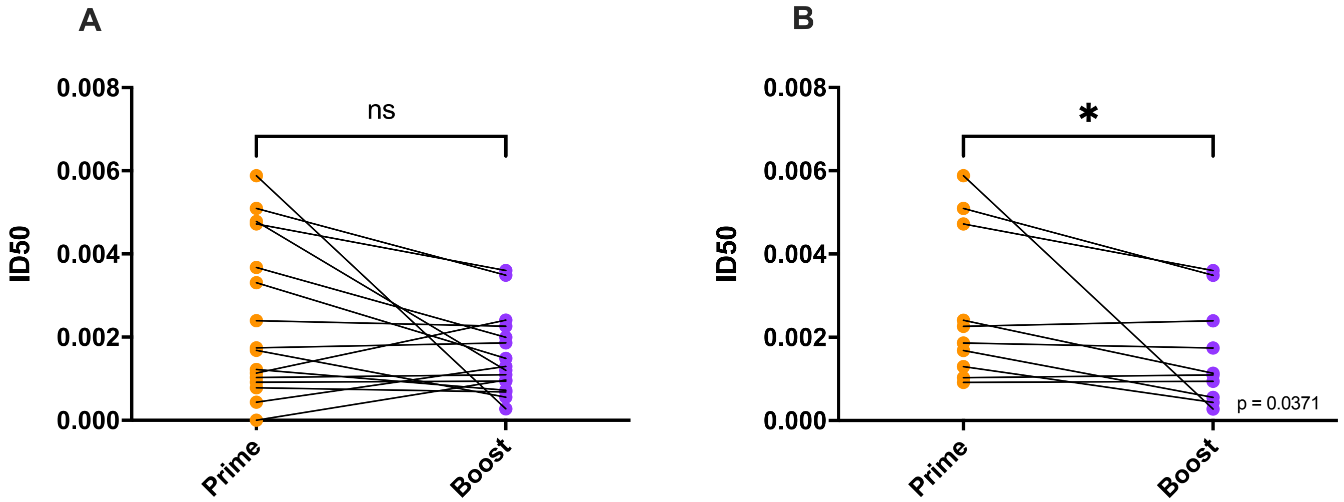

Sup. Fig. 8. ID50 comparison within matched samples BNT162b2 prime and boost. **A:** All BNT162b2 vaccinated samples. **B:** BNT162b2 vaccinated samples without a positive diagnosis for COVID-19 prior to vaccination. Wilcoxon paired matched rank test evidenced a significant decrease in ID50 post boost in samples without prior COVID-19 diagnostic.
